# The influence of antenatal imaging on prenatal bonding in uncomplicated pregnancies: a mixed methods analysis

**DOI:** 10.1186/s12884-024-06469-0

**Published:** 2024-04-11

**Authors:** Emily Skelton, Daniel Cromb, Alison Smith, Gill Harrison, Mary Rutherford, Christina Malamateniou, Susan Ayers

**Affiliations:** 1 Division of Radiography and Midwifery, School of Health and Psychological Sciences, City, University of London, London, EC1V 0HB UK; 2 Perinatal Imaging and Health, King’s College London, London, SE1 7EH UK; 3 Guy’s & St Thomas’ NHS Foundation Trust, London, SE1 7EH UK; 4 Society and College of Radiographers, London, SE1 2EW UK; 5 Centre for Maternal and Child Health Research, School of Health and Psychological Sciences, City, University of London, London, EC1V 0HB UK

**Keywords:** Attachment, Bonding, Fetal, Imaging, MRI, Parent, Ultrasound

## Abstract

**Background:**

Prenatal bonding describes the emotional connection expectant parents form to their unborn child. Research acknowledges the association between antenatal imaging and enhanced bonding, but the influencing factors are not well understood, particularly for fathers or when using advanced techniques like fetal magnetic resonance imaging (MRI). This study aimed to identify variables which may predict increased bonding after imaging.

**Methods:**

First-time expectant parents (mothers = 58, fathers = 18) completed a two-part questionnaire (QualtricsXM™) about their expectations and experiences of ultrasound (*n* = 64) or fetal MRI (*n* = 12) scans in uncomplicated pregnancies. A modified version of the Prenatal Attachment Inventory (PAI) was used to measure bonding. Qualitative data were collected through open-ended questions. Multivariate linear regression models were used to identify significant parent and imaging predictors for bonding. Qualitative content analysis of free-text responses was conducted to further understand the predictors’ influences.

**Results:**

Bonding scores were significantly increased after imaging for mothers and fathers (*p* < 0.05). MRI-parents reported significantly higher bonding than ultrasound-parents (*p* = 0.02). In the first regression model of parent factors (adjusted *R*^2^ = 0.17, *F* = 2.88, *p* < 0.01), employment status (β = -0.38, *p* < 0.05) was a significant predictor for bonding post-imaging. The second model of imaging factors (adjusted *R*^2^ = 0.19, *F* = 3.85, *p* < 0.01) showed imaging modality (β = -0.53), imaging experience (β = 0.42) and parental excitement after the scan (β = 0.29) were significantly (*p* < 0.05) associated with increased bonding. Seventeen coded themes were generated from the qualitative content analysis, describing how scans offered reassurance about fetal wellbeing and the opportunity to connect with the baby through quality interactions with imaging professionals. A positive scan experience helped parents to feel excited about parenthood. Fetal MRI was considered a superior modality to ultrasound.

**Conclusions:**

Antenatal imaging provides reassurance of fetal development which affirms parents’ emotional investment in the pregnancy and supports the growing connection. Imaging professionals are uniquely positioned to provide parent-centred experiences which may enhance parental excitement and facilitate bonding.

## Background

Ultrasound is used to evaluate fetal viability, development and well-being, and to identify occasions where medical intervention during pregnancy or shortly after birth may improve post-natal outcomes [1]. Yet, its efficacy as an imaging tool can be compromised by inherent limitations including fetal lie, maternal body habitus and operator technique [2]. As scan acquisition methods advance, fetal magnetic resonance imaging (MRI) has become popular to complement ultrasound in prenatal diagnosis because it provides increased anatomical detail for some physical conditions [3]. However, the imaging procedure is markedly different, and pregnant women and people may experience anxiety because of loud MRI machine noises, claustrophobia whilst in the MRI scanner, and discomfort in lying still for an extended period of time [4]. Compared to ultrasound examinations which do not usually exceed 30-min in duration, fetal MRI appointments may be scheduled for 60-min (although not all of this time is devoted to image acquisition) [5].

In addition to medical value, psychological benefits of fetal imaging are reported for expectant parents in providing an opportunity to see and connect with their unborn baby before birth [6]. For the non-pregnant parent, scans are also an opportunity to engage with the pregnancy and provide companionship and support to partners [7]. Broadly, parent-fetal bonding refers to the emotional connection that expectant parents feel towards their unborn babies during pregnancy [8]. This definition acknowledges the unidirectional nature of the parent-to-fetal relationship and considers the construct of bonding as theoretically distinct from original conceptualisations of attachment which are characterised by a system of care-seeking and care-giving behaviours after birth [9]. Quality prenatal bonding is associated with parental wellbeing and positive behaviours during pregnancy (e.g., smoking cessation) that subsequently contribute to healthy infant brain and neurological development [10]. Prenatal bonding is also thought to predict postnatal attachment [11], and further links between parent-fetal bonding (particularly the paternal-fetal relationship) and the child’s cognitive and socio-economic development also highlight the importance of studying this construct [12]. However, terminology of bonding and attachment are often used interchangeably to reflect varying definitions in the literature and as such, different approaches are utilised in attempts to evaluate not only the strength of the bond itself [9], but also the effect of interventions designed to facilitate its development [13]. Subsequently, inconsistent methodological approaches and varying quality in existing research studies have produced conflicting findings [14].

Fetal ultrasound images are thought to facilitate parents’ connection to the baby by providing visual knowledge that can be used to further enhance mental representations of the imagined child [11]. A recent literature review including 23 studies concluded that parent-fetal bonding was enhanced following antenatal imaging [6]. In particular, the role of the sonographer (a healthcare professional who performs ultrasound scans) in creating a parent-centred scan experience was highlighted as an important factor to facilitate bonding. Expectant parents rely on sonographers not only to assess fetal health, but also to transform the medical entity captured within the acquired images into relatable individuals who they can interact with, and place in their own realities [15]. MRI images, like ultrasound, are also dependent on expert clinical interpretation [16], however, they are less familiar to expectant parents than ultrasound, and there is little understanding of how parents respond to these highly detailed anatomical visualisations of their unborn baby [6].

MRI is not currently part of the routine fetal screening pathway in England [17], but is used for more complex clinical investigations where ultrasound is inconclusive, or in research studies aiming to improve understanding of human development. Although the images produced are considered higher quality because they are not affected by the previously described limitations associated with ultrasound, it is unlikely that it will replace it due to increased financial cost and limited availability of specialist fetal MR imaging services [16]. This means that many studies reporting expectant parents’ experiences and perceptions of MRI are set in the context of a prenatal diagnosis where increased parental anxiety and distress may be a moderator of bonding [4, 18]. They are also retrospective, therefore many variables or confounding factors are missing, or cannot be controlled for. Prospective research is required to further understand parental experiences and the potential influence of MRI on bonding. Additionally, research exploring the paternal-fetal bond is limited compared to maternal studies [19]. As fathers and partners are now increasingly involved in pregnancy [7], it is important to better understand their perceptions, experiences and individual needs around accessing antenatal care in order for services to be inclusive and supportive [20].

Based on other literature exploring bonding and scan experiences in pregnancy [6], it was hypothesised that parent-fetal bonding scores would increase after imaging. Therefore, this study aimed to further identify parental and scan variables which may be associated with enhanced parent-fetal bonding after ultrasound or MRI, and qualitatively explore how they may facilitate the developing connection.

## Methods

The STROBE checklist was used to guide reporting [21]. A two-part questionnaire was developed for data collection, hosted on the Qualtrics XM™ platform (www.qualtrics.com).

**Fig. 1:**
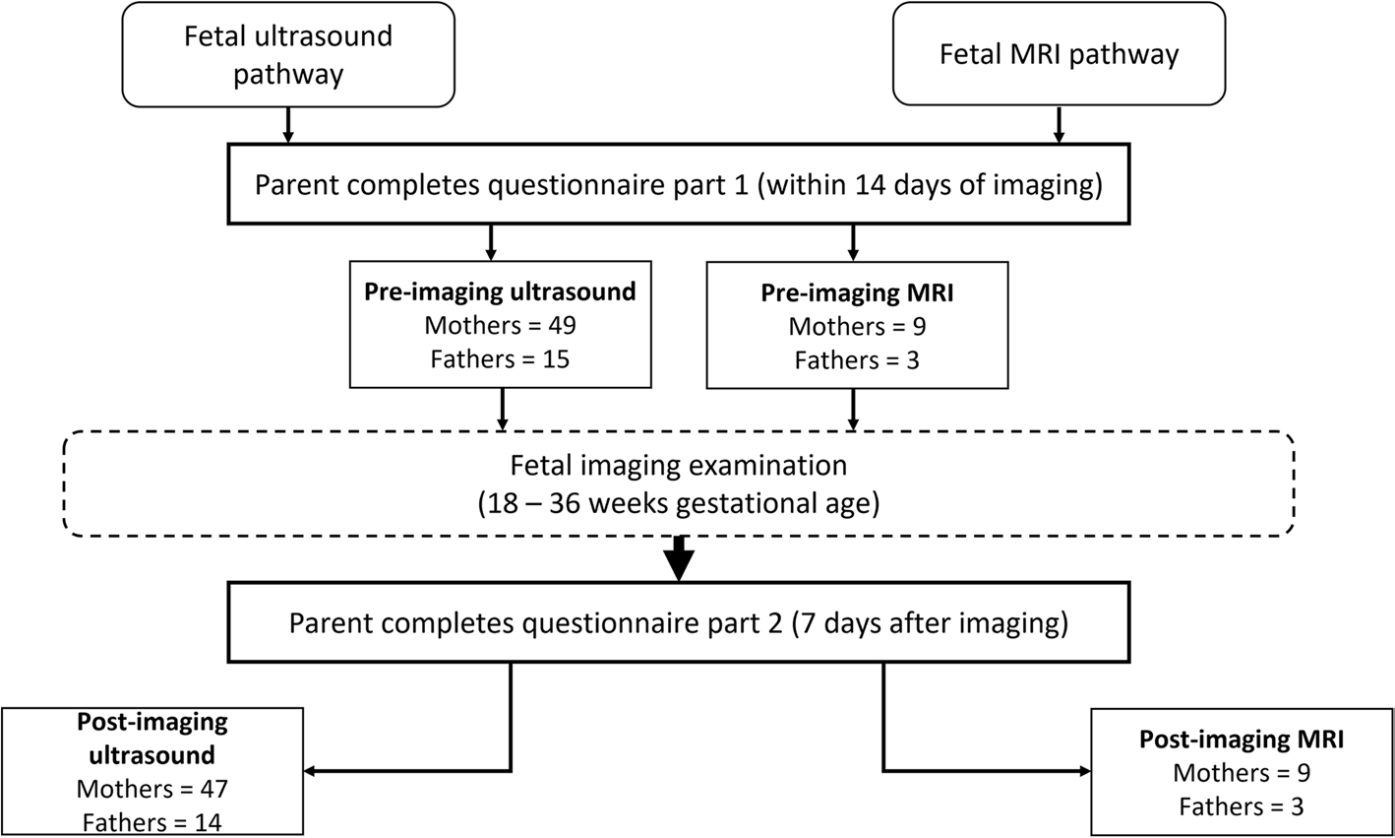
Schedule of participation

Recruitment ran between October 2021-December 2022. First-time expectant parents (≥ 18 years) attending a London hospital for fetal imaging (routine ultrasound or research MRI) between 18–36 weeks gestation in uncomplicated pregnancies were eligible to participate. Convenience sampling was used; ultrasound-parents were identified by clinical staff following completion of their routine first trimester screening scan between 11^+2^–14^+1^weeks of pregnancy [17], and MRI-parents were identified by perinatal imaging researchers when booking their research MRI scan. An introductory email was sent to prospective parents containing links to the participant information video and electronic informed consent form, which was designed according to good practice recommendations [22]. Once recruited, participants were allocated a unique identification number which they used to access the questionnaire. Two weeks before the imaging appointment, the weblink to part one of the questionnaire (pre-imaging) was shared. The link to part two (post-imaging) was shared one week after the scan (Fig. 1). Reminders to complete the relevant parts of the questionnaire were sent at 7 and 14 days after they were initially shared.

### Measures

**Fig. 2:**
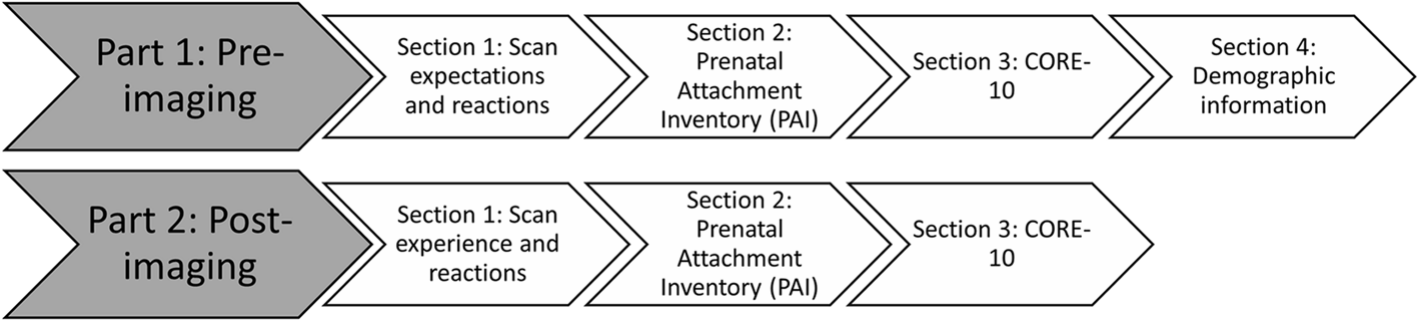
Questionnaire structure

Part one of the questionnaire contained four sections, and part two was composed of three (Fig. 2). Demographic information was only collected in the first part.

#### Prenatal attachment inventory (PAI)

A modified version of the Prenatal Attachment Inventory (PAI) [8] was used to measure parent-fetal bonding. Gendered items were removed or rephrased so that both mothers and fathers could respond to the same questions (e.g. “I tell others what the baby does inside me” became “I tell others what the baby does inside the womb”). For each item, parents were asked to select a Likert-response of “Almost Never,” “Sometimes,” “Often,” or “Almost Always.” A value between 1–4 was allocated to each response, and the total PAI score was calculated. Higher scores are associated with a more developed bond [23], and in this 16-item PAI, the maximum possible score was 64. Good reliability of the modified PAI is previously established [24, 25]. In this study, Cronbach’s alpha (α) was 0.90, indicating excellent internal consistency.

#### CORE-10

Psychological distress in participants was evaluated using the CORE-10 [26], which has been validated for use in the perinatal population [27]. Participants were asked to respond to 10-items using one of five Likert-responses ranging from “Not at all,” to “Most or all of the time” based on their experiences during the preceding week. Responses were allocated a value between 0–4 and combined. Total scores of ≥ 25 are associated with severe psychological distress [28]. Cronbach’s alpha (α) for the CORE-10 was 0.84.

#### Parental expectations, experience and reactions to antenatal imaging

**Fig. 3:**
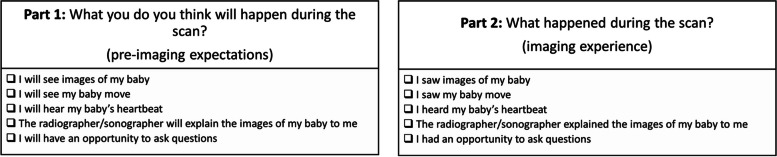
Matched questions to evaluate pre-imaging expectations and imaging experience

Exisiting measures of parental expectations and experiences of antenatal imaging [29] were not suitable for the current study’s focus on bonding so a measure was specifically developed for use based on prior literature findings and research studies [6, 25]. For statistical comparison, expectation and experience factors were matched (Fig. 3). An overall score was calculated from the total number of factors (maximum 5 score).

Rating scales (where 0 = not at all and 10 = extremely) were utilised for participants to report their reactions to imaging (anxiety and excitement prior to imaging, and anxiety, excitement, and satisfaction after imaging). Open-questions (e.g., What are you least looking forward to about your scan? What did you most enjoy about your scan?) were also included to further capture parental perspectives.

Representatives from UK-based support charities, Antenatal Results and Choices (ARC) and Fathers Reaching Out were invited to review the questionnaire and provide feedback regarding readability and usability. This resulted in minor amendments to the presentation (e.g., change of rating scale slider) for ease of use on a mobile device. Prior to launch, the questionnaire was piloted by parent volunteers (*n* = 7). The QualtricsXM™ platform contained instructions for navigating the questionnaire, including the use of directional tools to move between sections for editing. Participants could complete the questionnaire with no time limit enforced. The option to save and return to an incomplete questionnaire at a different time was also available.

### Data analysis

Sample size for paired analysis was informed by a power calculation based on previous studies evaluating the change in maternal–fetal bonding after antenatal ultrasound [30, 31]. From these, it was assumed that bonding scores may be increased by an average of 3-points. Using an alpha level of 0.05 and power of 80%, the minimum sample size required for this study was estimated as *n*= 70. Sample size for regression analysis was guided by published literature suggesting the number of subjects per independent variable should lie between 5–20 [32]. Therefore, it was aimed to include 10 subjects per variable in each model.

Quantitative data were analysed using Microsoft Excel (version 2008) and IBM SPSS Statistics (version 29). Frequencies and descriptive statistics including average scores for imaging expectations and experience, PAI and CORE-10 scores were calculated for each parent group. Kolmogorov–Smirnov tests indicated normally distributed data, therefore parametric statistical analyses were performed [33]. Independent and paired t-tests (assuming unequal variances where Levene’s statistic was significant) were used to compare means. Hedge’s g statistic (g) determined effect size. Cases were excluded from some analyses where paired data was unavailable. Two multivariate linear regression analyses were run to identify predictors significantly associated with enhanced bonding after imaging. Parent variables (e.g., parent demographics and social factors) were entered into the first model and scan variables (e.g., imaging modality, experience, and parental reactions) were entered into the second. Categorical variables were converted to binary-coded dummy variables (e.g., ethnicity became majority/white or minority/non-white) to enable their inclusion in the regression analysis whilst minimising the potential for overfitting in the models [34]. Statistical significance was determined at *p* < 0.05.

Qualitative content analysis of free-text responses was undertaken to help explain findings of the regression models. This was chosen over more interpretative methods because the brevity of responses was not conducive to deep analysis [35]. A deductive coding system was developed ES (a sonographer with 12 years’ experience in obstetric ultrasound) using the significant predictors identified in the regression models as coding clusters [36]. Responses were first organised into clusters and abstracted into units of meaning. Identified units were recontextualised and grouped into initial coded themes and reviewed against the original data. Coded themes were refined before being checked against the coding clusters to ensure their appropriate classification [37]. To evaluate reliability of the coding system, re-coding of a randomised 10% of the qualitative responses was independently performed by DC (a paediatrician and clinical research fellow with 5 years’ experience of fetal MRI). Following this, minor changes to the coding descriptors were made for improved clarity. Inter-coder agreement on 10% of the content was 96% following resolution of discrepancies.

### Ethics

Ethical approval was received by the West of Scotland REC 3 (REC reference: 20/WS/0132, date of approval 12th November 2020) and School of Health and Psychological Sciences REC at City, University of London (REC reference: ETH1920-1680, date of approval 30th November 2020). Due to the sensitive nature of this research, only participants who were committed to continuing their pregnancy were approached to participate. The potential risk of parental anxiety caused by taking part was low, however a contact list of perinatal mental health support resources was shared after completing part one of the questionnaire. An emergency referral pathway was developed in conjunction with the local perinatal mental health team to provide urgent support for parents who scored highly for psychological distress although its use was never required.

## Results

All parents stated they were either the mother or father of the baby. A total of 76 expectant parents (58 mothers, 18 fathers) completed part one of the questionnaire. Of these, 64 had ultrasound and 12 had fetal MRI. Sixteen sets of parents were in a couple. Three parents did not respond to the invitation to complete part two, resulting in paired data for 73 parents (56 mothers, 17 fathers).

Mean maternal age was 32 (range = 23–39), and mean paternal age was 34 (range = 28–41). Most parents were educated to postgraduate degree level (*n* = 39, 51.3%), of white ethnicity (*n* = 57, 75.0%) and in full-time employment (*n* = 64, 84.2%). Sixteen parents (21.1%) disclosed a pre-existing physical health condition, and twenty (26.3%) reported receiving a previous diagnosis of, or support for a mental health condition (Table 1).

**Table 1:** Participant characteristics

	**Ultrasound-Mothers**	**Ultrasound-Fathers**	**MRI-Mothers**	**MRI-Fathers**
**Questionnaire part 1**	*n* = 49	*n* = 15	*n* = 9	*n* = 3
**Questionnaire part 2**	*n* = 47	*n* = 14	*n* = 9	*n* = 3
**Mean age**	32.22 (SD = 3.15)	34.40 (SD = 3.76)	32.22 (SD = 3.96)	32.67 (SD = 2.89)
**Ethnicity**				
White	*n* = 34 (69.39%)	*n* = 13 (86.67%)	*n* = 8 (88.89%)	*n* = 2 (66.67%)
Mixed	*n* = 4 (8.16%)	*n* = 1 (6.67%)	*n* = 0	*n* = 1 (33.33%)
Black	*n* = 3 (6.12%)	*n* = 0	*n* = 0	*n* = 0
Asian	*n* = 5 (10.20%)	*n* = 0	*n* = 0	*n* = 0
Other	*n* = 3 (6.12%)	*n* = 1 (6.67%)	*n* = 1 (11.11%)	*n* = 0
**Education level**				
College (A-Levels)	*n* = 3 (6.12%)	*n* = 2 (13.33%)	*n* = 0	*n* = 0
Undergraduate degree	*n* = 15 (30.61%)	*n* = 6 (40.00%	*n* = 5 (55.56%)	*n* = 2 (66.67%)
Postgraduate degree	*n* = 29 (59.18%)	*n* = 5 (30.33%)	*n* = 4 (44.4%)	*n* = 1 (33.33%)
Doctorate	*n* = 2 (4.08%)	*n* = 2 (13.33%)	*n* = 0	*n* = 0
**Employment status**				
Full-time	*n* = 41 (83.67%)	*n* = 13 (86.67%)	*n* = 7 (77.78%)	*n* = 3 (100%)
Part-time	*n* = 4 (8.16%)	*n* = 2 (13.33%)	*n* = 0	*n* = 0
Student	*n* = 1 (2.04%)	*n* = 0	*n* = 0	*n* = 0
Unemployed	*n* = 2 (4.08%)	*n* = 0	*n* = 1 (11.11%)	*n* = 0
Other	*n* = 1 (2.04%)	*n* = 0	*n* = 1 (11.11%)	*n* = 0
**Mental health condition**				
Yes	*n* = 17 (34.69%)	*n* = 1 (6.67%)	*n* = 2 (22.22%)	*n* = 0
No	*n* = 30 (61.22%)	*n* = 14 (93.33%)	*n* = 6 (66.67%)	*n* = 3 (100%)
Prefer not to say	*n* = 2 (4.08%)	*n* = 0	*n* = 1 (11.11%)	*n* = 0
**Physical health condition**				
Yes	*n* = 12 (24.49%)	*n* = 1 (6.67%)	*n* = 3 (33.33%)	*n* = 0
No	*n* = 37 (75.51%)	*n* = 14 (93.33%)	*n* = 6 (66.67%)	*n* = 3 (100%)

Fetal imaging was performed between October 2021-December 2022. Mean gestational age (GA) in weeks and days at the time of the scan was 21^+1^ (range: 18^+6^–33^+2^) for ultrasound and 27^+1^ (range: 18^+4^–35^+4^) for MRI.

### Parent-fetal bonding (PAI)

Bonding was significantly increased in mothers (*p* < 0.001) and fathers (*p* = 0.04) after imaging. Mean increase was larger in mothers (4.71, g = -0.81) than fathers (3.06, g = -0.53). No significant differences in mean scores were observed between mothers and fathers pre or post-imaging (Table 2). MRI-parents had significantly higher bonding scores than ultrasound-parents, both before and after imaging. The pre-imaging mean difference in PAI was 7.25 (*p* = 0.01, g = -0.85). Post-imaging, the mean difference was 6.46 (*p* = 0.02, g = -0.74).

**Table 2:** Parent-fetal bonding (PAI pre and post-imaging (t-tests)

	**Pre-imaging PAI**	**Post-imaging PAI**	**Mean difference**	**t**	**Effect size (Hedge’s g)**
**All-mothers (paired data for *n* = 56)**^a^	38.02 (SD 8.47)	42.73 (SD 8.90)	4.71	-6.11^**^	-0.81
**All-fathers (paired data for *n* = 17)**^a^	35.53 (SD 8.45)	38.59 (SD 8.26)	3.06	-2.29^*^	-0.53
**Mothers vs. Fathers**	**All-mothers**	**All-fathers**	**Mean difference**	**t**	**Effect size (Hedge’s g)**
**Pre-imaging PAI**	38.19 (SD 8.41)	36.94 (SD 10.16)	-1.25	0.52	0.14
**Post-imaging PAI**	42.73 (SD 8.90)	38.59 (SD 8.26)	-4.14	1.71	0.47
**Ultrasound vs. MRI**	**All-ultrasound**	**All-MRI**	**Mean difference**	**t**	**Effect size (Hedge’s g)**
**Pre-imaging PAI**	36.75 (SD 8.77)	44.00 (SD 6.21)	7.25	-2.73^*^	-0.85
**Post-imaging PAI**	40.70 (SD 8.86)	47.14 (SD 7.04)	6.46	-2.38^*^	-0.74

^*^Significant at *p* < 0.05

^**^Significant at *p* < 0.001

^a^Participants with unmatched data were excluded from these analyses

### Predictors of bonding after imaging

Eight parent variables were entered into the first multivariate regression model (Table 3). This model was significant (adjusted *R*^2^ = 0.17, *F* = 2.88, *p* < 0.01) and showed that employment status was significantly predictive of parent-fetal bonding after imaging (β = -0.38, *p* < 0.05), with unemployed and part-time working parents scoring higher on the PAI than those in full-time work.

**Table 3:** Multivariate linear regression model for parent variables predicting post-imaging bonding

Independent variable	Standardised coefficient (β)
**Step 1: demographics**	
Parent type (mother or father)	0.17
Parent age	-0.06
Ethnicity (majority or minority)	0.20
**Step 2: social factors**	
Education (school or higher education)	0.02
Employment status (full-time or not)	-0.38^*^
**Step 3: physical and mental health**	
Mental health condition	0.08
Physical health condition	-0.24
Post-imaging CORE-10	-0.01
**Model summary**	*F* = 2.88 (*p* < 0.01) Adjusted *R*^2^ = 0.17

^*^Significant at *p* < 0.05

The second model was also significant (adjusted *R*^2^ = 0.19, *F* = 3.85, *p* < 0.01). Three of the six imaging variables (Table 4) were significantly predictive of bonding. These were imaging modality type (β = -0.53, *p* < 0.05), imaging experience (β = 0.42, *p* < 0.05), and parental excitement after imaging (β = 0.29, *p* = 0.02). Issues of multicollinearity were not indicated as variance inflation factors in the models were between 1.12–2.33 (tolerance = 0.54–0.87).

**Table 4:** Multivariate linear regression model for scan variables predicting post-imaging bonding

Independent variable	Standardised coefficient (β)
**Step 1: ultrasound or MRI**	
Imaging modality	-0.53*
**Step 2: scan factors**	
GA at scan	-0.06
**Step 3: experience**	
Imaging experience	0.42*
**Step 4: post-imaging reactions**	
Excitement	0.29*
Anxiety	0.09
Satisfaction	-0.15
**Model summary**	*F* = 3.85 (*p* < 0.01) Adjusted *R*^2^ = 0.19

^*^Significant at *p* < 0.05

### Parental expectations, experience, psychological distress and reactions to imaging

#### Pre vs. post-imaging

Average CORE-10 scores in all parents (including those with a prior mental health condition) were < 10 which indicated low-level psychological distress (not of clinical concern). Mothers’ pre and post-scan CORE-10 scores were similar, however fathers’ scores were significantly decreased after imaging (*p* < 0.001). Anxiety significantly decreased after imaging in mothers (*p* < 0.001) and fathers (*p* = 0.01). Fathers’ post-imaging excitement was significantly higher (*p* = 0.01), although this increase was not observed in mothers. No significant difference between pre-scan expectation and post-scan experience score was noted for mothers or fathers (Table 5).

**Table 5:** Parental expectations, experience, psychological distress and reactions to imaging

**All mothers (paired data for *n* = 56)**	**Pre-imaging**	**Post-imaging**	**Mean difference**	**t**	**Effect size (Hedge’s g)**
**Anxiety**	4.30 (SD 2.42)	2.14 (SD 2.34)	-2.16	7.17^**^	0.95
**Excitement**	7.38 (SD 2.01)	7.63 (SD 2.29)	0.25	-0.78	-0.10
**CORE-10**	8.86 (SD 5.00)	8.38 (SD 5.94)	0.48	0.94	0.12
**Expectations (pre-imaging) or experience (post-imaging)**	4.54 (SD 0.87)	4.61 (SD 0.62)	0.07	-0.66	-0.09
**All fathers (paired data for *n* = 17)**	**Pre-imaging**	**Post-imaging**	**Mean difference**	**t**	**Effect size (Hedge’s g)**
**Anxiety**	2.41 (SD 1.33)	1.47 (SD 0.94)	-0.94	2.89^*^	0.67
**Excitement**	7.47 (SD 2.50)	9.12 (SD 1.05)	1.65	-2.75^*^	-0.63
**CORE-10**	6.88 (SD 3.74)	4.82 (SD 3.01)	2.06	4.15^**^	0.96
**Expectations (pre-imaging) or experience (post-imaging)**	4.65 (SD 0.70)	4.53 (SD 1.01)	0.12	0.46	0.11

^*^Significant at *p* < 0.05

^**^Significant at the level *p* < 0.001, parental satisfaction was only rated post-imaging

#### Mothers vs. fathers

Although mean values suggest low anxiety in both parents, it was still significantly (*p* < 0.001) higher in mothers (4.21, SD = 2.45) compared to fathers (2.39, SD = 1.29) pre-imaging. Post-imaging, the mean difference between fathers’ (9.12, SD = 1.05) and mothers’ excitement (7.63, SD = 2.29) was also significant (*p* < 0.001). Fathers’ post-imaging satisfaction (9.12, SD = 1.05) was also significantly higher than mothers’ (8.36, SD = 1.78) although the effect size was small (*p* = 0.04, g = 0.46). A final significant difference (*p* = 0.02) was noted between mothers’ and fathers’ post-imaging CORE-10 scores, with mothers scoring higher (8.38, SD = 5.94) than fathers (4.82, SD = 3.09). No significant differences in pre-imaging excitement, pre-imaging CORE-10 or post-imaging anxiety were observed between mothers and fathers (Table 6).

**Table 6:** Parental expectations, experience, psychological distress and reactions to imaging

	**All mothers**	**All fathers**	**Mean difference**	**t**	**Effect size (Hedge’s g)**
**Pre-imaging:**					
**Anxiety**^a^	4.21 (SD 2.45)	2.39 (SD 1.29)	-1.82	4.11^**^	0.81
**Excitement**	7.38 (SD 2.03)	7.50 (SD 2.43)	0.12	-0.21	-0.60
**CORE-10**	8.64 (SD 5.07)	7.17 (SD 3.82)	-1.47	1.13	0.30
**Expectations**	4.55 (SD 0.86)	4.56 (SD 0.78)	0.01	-0.02	-0.00
**Post-imaging:**					
**Anxiety**^a^	2.14 (SD 2.34)	1.47 (SD 0.94)	0.67	1.74	0.32
**Excitement**^a^	7.63 (SD 2.29)	9.12 (SD 1.05)	1.49	-3.74^**^	-0.71
**Satisfaction**^a^	8.36 (SD 1.78)	9.12 (SD 1.05)	0.76	-2.18^*^	-0.46
**CORE-10**	8.38 (SD 5.94)	4.82 (SD 3.09)	-3.55	2.36^*^	0.65
**Imaging experience**	4.61 (SD 0.62)	4.53 (SD 1.01)	-0.08	0.39	0.11

^*^Significant at *p* < 0.05

^**^Significant at *p* < 0.001

^a^Equal variances not assumed, parental satisfaction only rated post-imaging

#### Ultrasound vs. MRI

There were very few differences between parents who had ultrasound or MRI. Ultrasound-parents had significantly higher pre-imaging expectation scores than MRI-parents (*p* = 0.01). Imaging experience scores between the modalities were also significantly different (*p* = 0.01), with ultrasound-parents scoring higher (4.75, SD = 0.47) than MRI-parents (3.75, SD = 1.14). No significant differences were observed between mean scores for anxiety, excitement, post-imaging satisfaction or CORE-10 in ultrasound-parents compared to MRI-parents.

### Qualitative findings

Of the four statistically significant predictors, qualitative data relating to parental employment were not collected, therefore this was not included as a coding cluster in the content analysis. A fourth category (parent type) was developed to further explore perspectives of mothers and fathers. Seventeen coded themes were generated (Table 7), representing 78.05% of the content. Coded themes are presented by statistical importance as per the regression analyses.

**Table 7:** Final clusters and coding themes

Coding cluster	Coded theme	Description	Quotations	Frequency of occurrence (n)	Percentage of free-text content (%)
**Imaging modality**	MRI as an advanced fetal imaging modality	Parents viewed fetal MRI as superior to US	*“We’d had ultrasound scans before, but I wasn’t prepared for how amazingly detailed the MRI would be.”*	13	1.04%
**Imaging experience**	Scans for confirmation of fetal health	Pregnancy scans considered a tool for reassurance about the pregnancy	*“I’m most looking forward to the confirmation that the pregnancy remains low risk and that all is as expected.”*	174	12.94%
	The potential for unexpected news	Parents felt anxious about the possibility of receiving unexpected news from the scan	*“I’m just worried that something is wrong with the baby and I get bad news.”*	111	8.12%
	Satisfaction with the scan experience	Parents felt satisfied with their scan experience when their expectations for care were adequately met	*“I do not think there are any improvements to be made. I was kept informed, well prepared and made comfortable.”*	84	6.19%
	Interaction with healthcare professionals	Parental experiences were supported by good communication during the scan to alleviate anxiety	*“The sonographer was professional but warm, sharing her expertise in an accessible and calm-inducing way. She talked through every step of the scan, the image, and also what might take a bit longer to see (but that didn’t mean there was a problem with the fetus) – i.e. managing our anxiety pre-emptively.”*	67	4.40%
	Facilitating improved care experiences	Parent-centred care requires sufficient time to experience the scan and ask questions	*“Unfortunately, they made it feel rushed and it’s such a huge moment for us that it felt like we were another tick off the list.”*	65	4.63%
	Uncomfortable scans	Despite some maternal discomfort, both scan procedures were tolerated because of their potential benefits	*“It was quite uncomfortable [in the MRI scanner], but of course I would get all necessary scans if there any concerns about my baby’s health.”* *“It was a little bit uncomfortable at one point when the baby’s head was very low down and the sonographer was trying to get the right angle to see the face clearly.”*	37	2.92%
	Attending hospital during a pandemic	Infection control measures were stressful for parents, especially as they were not supportive of partners’ attendance	*“It made everything harder to wait outside the hospital […] it made the entire process far more stressful and unpleasant. Pregnancy is not just for women – partners need to be involved at all times.”*	37	2.47%
	Scan extras	Some parents wanted more from their scans than can be offered in practice	*“I would love to have seen everything in 3D.”*	25	1.63%
	The unknowns of pregnancy scanning	Some parents did not feel fully prepared for scans	*“It took a long time and I wasn’t aware upfront exactly how long [it would take].”*	19	1.15%
**Parent excitement**	Seeing baby for oneself	Parents reassured by seeing their babies and enjoy the visual interaction	*“It was a lovely period of time to get to spend looking at the baby and seeing how they’re growing. It reassured me that everything was progressing as it should be.”*	197	13.43%
	Knowing baby’s sex	Parents excited to learn the fetal sex as it helped to personify their baby	*“I would have welcomed a girl or boy equally, but finding out the gender helps me visualise the reality of the baby coming into our lives…”*	78	3.77%
	Becoming a parent for the first time	The scan as a catalyst for the transition to parenthood	*“The scan completely changed how I felt about my baby…I definitely prioritised my baby more – my prior concerns about my career just don’t seem as pressing now.”*	66	4.77%
	Scans for bonding	The scan experience helped parents to feel more connected to their babies and each other	*“This scan really helped us to be in a better place mentally and to bond with our baby.”*	31	1.84%
**Parent type**	Fathers’ excitement to be involved	Scans provided an opportunity for fathers to be involved in the pregnancy	*“Being a Dad-to-be in this process, the scan is the only time I get to see the baby and interact with it.”*	62	4.78%
	Tentative motherhood	Mothers withheld excitement until they felt reassured by their scans	*“Having confirmation that the baby was growing as normal allowed me to be more excited and start to believe properly that the baby would be born without complications…”*	41	3.05%
	Mothers’ responsibility for scans	Mothers felt a greater sense of responsibility for the scan outcome	*“The baby wouldn’t move […] this was stressful as I felt I needed to do something about it […] but was unable to make him move. I felt like I’d done something wrong.”*	13	0.92%

#### Imaging modality

MRI-parents perceived the imaging technique as superior to ultrasound, however, in contrast to its importance in the regression analysis, it was not a high frequency theme in their open-text responses (*n* = 13, 1.04%).

#### Imaging experience

Parents regarded imaging as a tool to provide reassurance about fetal health (*n* = 174, 12.94%), although they were simultaneously anxious of the potential to receive unexpected news about a fetal anomaly or pregnancy complication (*n* = 111, 8.12%). Satisfaction in the experience was reported by parents who had their expectations for care adequately met (*n* = 84, 6.19%), which included feeling informed about the scan procedure. This was facilitated by positive interactions with radiographers and sonographers (*n *= 67, 4.40%), although the rushed “conveyor belt” experience was also described by some parents and identified as an area to address for improved provision of parent-centred care (*n* = 65, 4.63%). Discomfort in the scan procedure was reported for both modalities (*n* = 37, 2.92%). Ultrasound-mothers were uncomfortable because of transducer pressure on their abdomen, particularly if the fetal lie was unfavourable, and being scanned with a full bladder. MRI-mothers noted feelings of claustrophobia, loud scanner noises, and lying still for an extended period as causes of discomfort. Parental dissatisfaction was expressed in relation to hospital waiting times and COVID-19 infection control measures which were unsupportive of partner attendance (*n* = 37, 2.47%), as well as a lack of information about the scan (*n* = 19, 1.15%). Increased options for imaging extras including choosing souvenir photos, recording video clips, having 3-Dimensional ultrasound offered as standard, and receiving MRI images immediately after the scan were suggested as further means to improve experiences (*n* = 25, 1.63%).

#### Parent excitement

References to “seeing baby” were most frequently observed in the free-text responses (*n* = 197, 13.43%). Parents enjoyed visualising fetal movement and cardiac activity during scans as it provided reassurance. Images helped parents to personify the fetus, creating a sense of familiarity that could be further intensified by learning the fetal sex (*n* = 78, 3.77%). For some parents, the scan marked a pivotal moment to accept the reality of pregnancy and embrace the transition to parenthood (*n* = 66, 4.77%). The scan experience was perceived by both parents as beneficial, particularly for fathers in enhancing their emotional connection with the baby, and strengthening the partner relationship (*n* = 31, 1.84%).

#### Parent type

Many parents reported that in the absence of any physical experience of pregnancy, imaging provided a unique and exciting opportunity for fathers’ engagement (*n* = 62, 4.78%). Mothers reported greater apprehension prior to scans due to the possibility of an unexpected finding, and actively supressed excitement until receiving confirmation of fetal health (*n* = 41, 3.05%). Mothers’ anxiety was also created by assuming greater responsibility for the scan or pregnancy outcome (*n* = 13, 0.92%), for example fetal sex or position.

## Discussion

In this study, parent-fetal bonding scores were significantly increased following imaging in both parents which is consistent with existing literature [6]. However, in contrast to other studies [25, 38–40], bonding scores were not observed to be significantly different between mothers and fathers. Four variables were identified as significant predictors of parent-fetal bonding after imaging: scores were significantly higher in parents who had MRI, who scored their imaging experience and excitement levels higher, and who were not in full-time employment. Parental excitement in visualising their baby and the positive experience of receiving confirmation of fetal health were the most frequent references in the qualitative content analysis.

### Interpretation

Many parents regarded imaging as a tool for reassurance of fetal development and wellbeing, and, mothers in particular, described how they attempted to supress excitement about the pregnancy until receiving confirmation of fetal health [41]. Whilst it has been suggested that conceptualisations of the “tentative pregnancy” may indicate detachment from the fetus in parents’ reluctance to embrace the developing bond [42], it has been argued that this response (often perceived as anxiety or worry about a possible unexpected physical condition or pregnancy loss) actually demonstrates the presence of this connection as fear that the imagined baby may not become reality [14].

The high frequency of references made to ‘seeing baby’ shows how scans provided powerful visual evidence used by parents to further validate assurances of fetal health offered by healthcare professionals [43]. However, in addition to reassurance, the images could be regarded as a source of uncertainty, creating anxiety if parents are not guided in how to interpret them [1]. Further uncertainty may also be created by communication around the limitations of prenatal screening [44], particularly if acquired images are low-quality [2]. Anxiety was significantly decreased for both parents after imaging, suggesting scans helped to mitigate this reaction. Additionally, some parents may not identify as anxious before the scan, however, expressing relief post-imaging may imply suppressed anxiety [29]. It has been suggested that the need for reassurance arises from anxiety created by the scan itself and uncertainties in fetal screening [45]. This may partly explain why parents perceived MRI as superior, due to its reputation as a more objective, diagnostic modality [46]. The wider field-of-view also enables parents to visualise the whole fetus instead of a series of 2-Dimensional cross-sectional images. However, as with ultrasound, MRI images require skilful interpretation, which is dependent on a clinician’s specialist knowledge and experience [16], therefore it may not actually be considered completely objective.

Other explanations may be offered to further understand the association between MRI and higher bonding scores. First, it could be argued that as these scans occurred at a more advanced GA (and these parents would have already received reassurance about fetal health from routine ultrasound screening scans) their emotional connection was more developed [47]. However, although higher MRI bonding scores were consistently noted compared to ultrasound, GA was not found to be a significant predictor in the regression analysis. Secondly, it must be acknowledged that unlike ultrasound, MRI scans were performed for research purposes. Parents may volunteer for pregnancy research because of its perceived benefits to the fetus [48], which suggests emotional investment through demonstration of responsible parenting [49]. Alternatively, parents experiencing a deeper connection may have been more motivated at the opportunity to see their baby again [50].

The findings also suggest how parental excitement is increased after imaging, and why this may help to enhance bonding. Parents reported feeling excited to ‘see the baby’ and ‘hear the heartbeat’. Visual and audial scan cues may substantiate fetal presence, and facilitate growing tangibility of the baby [14]. After scanning, some parents remarked how the pregnancy felt more ‘real’ and expressed excitement imagining the baby in their lives. This may highlight scans as a’trigger moment’ where the bond is initiated or intensified [19], and parents are prompted to engage with their new caregiving role [51]. For some, the scan was an opportunity to learn the fetal sex, which further contributed to feelings of closeness to the baby and excitement. Yet, it has been argued that knowing the fetal sex may actually be problematic for bonding [14], particularly if it does not align to parental preferences, or is inaccurate, as this mismatch in expectations requires parents to adjust their existing mental depictions [52].

Regardless of imaging modality, fathers’ excitement was noted to be consistently and significantly higher than mothers. Whilst some free-text responses alluded to fathers lack of awareness or anxiety for unexpected news to explain this [53], it may also be considered that fathers were increasingly excited about the opportunity to be involved in an aspect of antenatal care [7]. Fathers and partners are more likely to attend ultrasound scans than other antenatal checks [54]. Nevertheless, being present does not guarantee a positive experience for either parent, especially if healthcare professionals fail to fully acknowledge the partner’s role [55]. Pregnancy is regarded as a psychologically demanding time for fathers transitioning into their parental role [56], and conflicting emotions experienced during this time may be associated with feelings of chaos or loss of control [57].

It has been suggested that healthcare professionals are not adequately trained to engage with partners [58] which leads to their exclusion from care interactions [59, 60] and further contributes to feelings of confusion and isolation [61]. In this study, COVID-19 infection control measures in the ultrasound department requiring fathers to wait in a separate area of the hospital to their partners created stress for both parents. This reflects findings reported in relation to the COVID-19 pandemic when partners were temporarily restricted from attending scans [25]. As they do not physically experience pregnancy, providing support through companionship is thought to be a key aspect of how expectant fathers conceptualise their role during the prenatal period [19]. Being unable to fulfil this role reinforces feelings of inadequacy, which can negatively affect the sense of connection to the pregnancy [62]. Partner inclusion is important for prenatal bonding and to support maternal emotional wellbeing [63], therefore, healthcare professionals should make efforts to involve partners by acknowledging the importance of their presence [57], providing father-focused information [20], and directing conversation to both parents [64]. ‘Interactions with healthcare professionals’ was developed to highlight the integral role of the imaging professional in facilitating good communication, which contributed to positive parental experiences and reduced anxiety. Thoroughly explaining the scanning process and images, being open to questions and not rushing through the appointment were identified as central to parent-centred care. Indeed, previous literature has reported improved satisfaction in the scan experience associated with increased feedback from healthcare professionals [29, 65]. However, recent research suggests that moral injury and occupational burnout experienced by UK obstetric sonographers because of the COVID-19 pandemic may present substantial challenges to the provision of parent-centred care [66, 67].

Whilst the influence of parental employment (e.g., unemployed or part-time working) to enhance bonding was not further qualified, it may be that parents in full-time employment have reduced cognitive capacity to engage in imaginative practices which are essential to facilitate the developing bond, as they may be preoccupied with procedural and operational aspects of their work [68]. A similar explanation relating to cognitive capacity was proffered pertaining to the negative effect of anxiety related to COVID-19 pandemic on parent-fetal bonding [69], where it was argued that increasing preoccupation with pandemic-related anxiety in mothers decreased their capability to think about the baby [70].

### Clinical implications

Although various scales attempt to quantify parent-fetal bonding [71], the clinical use of this metric is uncertain. Whilst higher scores are typically considered to reflect a more developed bond, no optimal value has been reported [23]. A positive correlation between bonding and GA has been previously observed [72], and supports the theory of key ‘trigger moments’ throughout pregnancy to intensify the bond [19]. However, this implies that bonding is a linear process, which may not be reflective of all parents’ experiences. Instead, it has been suggested that even if ‘low’ bonding scores are recorded by parents earlier in the pregnancy, their developing connection is likely to be comparable with other parents at the end of the pregnancy [14]. As such, it is possible to inaccurately label a prenatal bond as dysfunctional, which may cause expectant parents to feel inadequate, and thus have substantial implications, not only for the developing bond, but postnatal infant attachment [73]. In addition, it may be argued that the development of an optimal value based on self-reported scores would not adequately reflect the theoretical complexity of the prenatal bonding construct, and therefore should not be considered in isolation to guide the provision of enhanced support for expectant parents. Thus, it is recommended in the first instance that a parent-centred approach to care which recognises and meets the individual needs of expectant parents is adopted within fetal imaging services to facilitate supportive experiences that may, in turn, promote enhanced parent-fetal bonding. Indeed, studies reporting the positive effect of healthcare consultations on prenatal bonding further reflect the findings of this study [74, 75], and suggest that the care interaction experienced during fetal imaging may be an important moderator to consider in the antenatal setting [76].

### Strengths and limitations

Prospective data collection facilitated engagement with different parent groups and modalities to enable focused comparisons to be made. Additionally, many studies evaluating parent-fetal bonding after imaging are purely quantitative; in this study, free-text responses provided qualitative context to extend the statistical findings [77]. A further strength was the use of validated instruments for data collection in all parents which permitted direct comparisons to be made between parent groups. However, self-reported bonding scores may be limited by social desirability bias [78]. In this context, parents completing the questionnaire may have altered their responses to achieve a higher score [73]. It has also been suggested that fathers may not disclose negative feelings if they think doing so may detract professional care and attention from their partner, or if they do not believe they are entitled to [79]. Another limitation was the predominance of ultrasound-mothers in the sample. Lack of fathers’ engagement in pregnancy research is acknowledged [80], and despite targeted efforts to recruit fathers into this study, numbers are low, reflecting the need to further improve approaches. In addition, recruitment of eligible MRI-parents was affected by continued disruption of research studies after the peak of the COVID-19 pandemic [81]. Although the pre-determined target sample size of *n*= 70 was achieved, it is likely that a greater number of participants would provide further power in the quantitative findings [82]. However, it should be noted that in addition to the challenges experienced in recruiting fathers into antenatal research, as a relatively new imaging modality in pregnancy, the provision of fetal MRI in the UK is limited. Thus, these initial findings serve to provide preliminary insight into expectant parents’ experiences of this technology and future work should seek to build on this. Enlarging the dataset and extending the sample population would also be beneficial to include greater representation of parents (including same-sex couples or non-binary parents), ethnicities and educational level.

## Conclusions

A detailed understanding of the influence of antenatal imaging on the developing parent-fetal bond is essential to ensure the provision of supportive and inclusive care for expectant parents accessing imaging services. This work extends existing knowledge by directly comparing mothers and fathers, and introduces new insights related to the use of fetal MRI in uncomplicated pregnancies. Bonding scores were significantly increased in both parents after imaging, however no differences between mothers and fathers were observed. Bonding was greater in parents after MRI compared to ultrasound although this may reflect the more developed emotional connection at later GAs. Parental excitement and experience were also identified as important variables, and qualitative analysis suggested they may be influenced by the professional conduct of imaging professionals during the scan. Effective communication helped parents to interpret scan images and offered reassurance of fetal wellbeing, contributing to a positive experience. Visualisation of the fetus provided evidence of its presence, which intensified parents’ sense of connection to the baby and increased excitement in imagining future parenthood. Imaging professionals should therefore adopt an informed, parent-centred approach to care to best support expectant parents.

## Data Availability

There are ethical restrictions on public sharing of this study’s dataset because of limited anonymity. However, a minimum dataset will be made available on reasonable request to the lead author (emily.skelton@city.ac.uk) and institutional research ethics committee (researchethics@city.ac.uk).

## Abbreviations

GA: Gestational age
MRI: Magnetic resonance imaging
PAI: Prenatal attachment inventory
